# An Investigation of Processes for Glass Micromachining

**DOI:** 10.3390/mi7030051

**Published:** 2016-03-22

**Authors:** Nguyen Van Toan, Masaya Toda, Takahito Ono

**Affiliations:** 1 Microsystem Integration Center (μSIC), Tohoku University, Sendai 980-8579, Japan; 2 Graduate School of Engineering, Tohoku University, Sendai 980-8579, Japan; mtoda@nme.mech.tohoku.ac.jp (M.T.); ono@nme.mech.tohoku.ac.jp (T.O.)

**Keywords:** glass micromachining, wet etching, sandblast, reactive ion etching, glass reflow process

## Abstract

This paper presents processes for glass micromachining, including sandblast, wet etching, reactive ion etching (RIE), and glass reflow techniques. The advantages as well as disadvantages of each method are presented and discussed in light of the experiments. Sandblast and wet etching techniques are simple processes but face difficulties in small and high-aspect-ratio structures. A sandblasted 2 cm × 2 cm Tempax glass wafer with an etching depth of approximately 150 µm is demonstrated. The Tempax glass structure with an etching depth and sides of approximately 20 μm was observed via the wet etching process. The most important aspect of this work was to develop RIE and glass reflow techniques. The current challenges of these methods are addressed here. Deep Tempax glass pillars having a smooth surface, vertical shapes, and a high aspect ratio of 10 with 1-μm-diameter glass pillars, a 2-μm pitch, and a 10-μm etched depth were achieved via the RIE technique. Through-silicon wafer interconnects, embedded inside the Tempax glass, are successfully demonstrated via the glass reflow technique. Glass reflow into large cavities (larger than 100 μm), a micro-trench (0.8-μm wide trench), and a micro-capillary (1-μm diameter) are investigated. An additional optimization of process flow was performed for glass penetration into micro-scale patterns.

## 1. Introduction

Due to the superior material properties of glass, including transparency, mechanical robustness, and dielectric properties, the glass has been widely used for micro-nano mechanical systems [1,2], micro-nano fluidic devices [3,4], and optical MEMS (Microelectromechanical systems) devices [5]. The glass substrate can be easily joined to a silicon substrate via the anodic bonding process without any additional adhesive, whereas these bond seals show good hermetic vacuum [6,7] and high bonding strength [8]. Unfortunately, glass is not easy to be machined precisely in a micro-nano scales.

Micro-nano fabrication technologies of silicon have been studied and well developed over the last several decades. The patterning silicon structures with high aspect ratios can be easily achieved via deep RIE techniques [9]. In turn, glass micromachining is much less investigated. There are many studies on the etching of SiO_2_ [10,11,12]; nevertheless, the etching of glass has more difficulties than that of SiO_2_. The components of Tempax glass consist of approximately 75% SiO_2_, 13% Na_2_O, 10.5% CaO, and other minor additives such as 1.3% Al_2_O_3_, 0.3% K_2_O, *etc*. Therefore, the glass is not pure SiO_2_, but other compositions that have a different etch rate in the etching processes are added to it. Thus, low aspect ratio, low etching rate, limited mask selectivity, and high surface roughness are still current problems in glass micromachining.

Several techniques of glass micromachining currently exist, including drilling [13], milling [13], laser [13], sandblasting [13], wet etching [14,15], dry etching [16,17,18], glass molding techniques [6,19,20,21,22], *etc*. The first three methods are usually used for quite large pattern sizes and face problems with small structures. The sandblast technique leads to a rough etching surface and has difficulty in the fabrication of small patterns below 100 μm. The wet etching of deep glass etching can be achieved with smooth sidewalls; however, due to its isotropic etching behavior the aspect ratio is limited. Moreover, the dimension reproducibility may be difficult with respect to side-etching. In contrast to dry etching, it can realize precise micromachining; however, the challenges of etching rate, mask selectivity, and etch quality in deep etching remain. Glass molding techniques, also known as glass blowing and glass reflow, are potential techniques for wide range microsystem applications. In essence, the glass blowing can be thought of as the reverse of the glass reflow. Glass blowing has been described in previous publications [19]. First, an etched cavities in silicon is bonded with thin glass wafer. Then, this wafer is heated inside a furnace at a high temperature. Due to the expansion of the trapped gas in the cavities, the glass is blown into three-dimensional spherical shells. High *Q* factor micro-glass-blown wineglass resonators have been presented [20]. Additionally, double-sided micro-lens array have been successfully demonstrated using glass blowing [21]. In turn, for glass reflow, vacuum cavities are required. The vacuum applies a force on the glass within the vacuum cavities, pulling it into the cavities during the high temperature process. Glass can penetrate into the large cavity; however, the glass is not easily pulled into a narrow pattern [6,22].

In this work, four techniques for glass micromachining are investigated and evaluated, including sandblast, wet etching, RIE, and glass reflow techniques. Sandblast and wet etching techniques are simple processes but they face difficulties with small and high-aspect-ratio structures. Deep Tempax glass pillar structures with smooth surfaces, vertical shapes, and high aspect ratios by using RIE are also studied. Finally, glass reflow into large cavities, a micro-trench and a micro-capillary is investigated.

## 2. Experiments and Discussions

### 2.1. Sandblast

Sandblasting is a technique in which a particle jet is directed towards a target (sample) for material removal by mechanical erosion via the impingement of high velocity abrasive particles. The sandblast process can be used for etching various materials such as glass [13], ceramics [2] (example: LTCC (low temperature co-fired ceramics)), and silicon.

**Figure 1:**
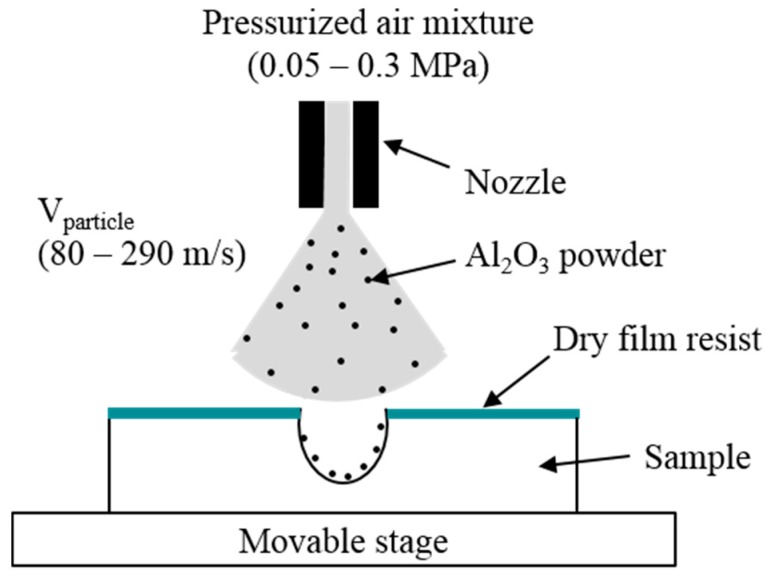
Sandblast setup.

**Figure 2:**
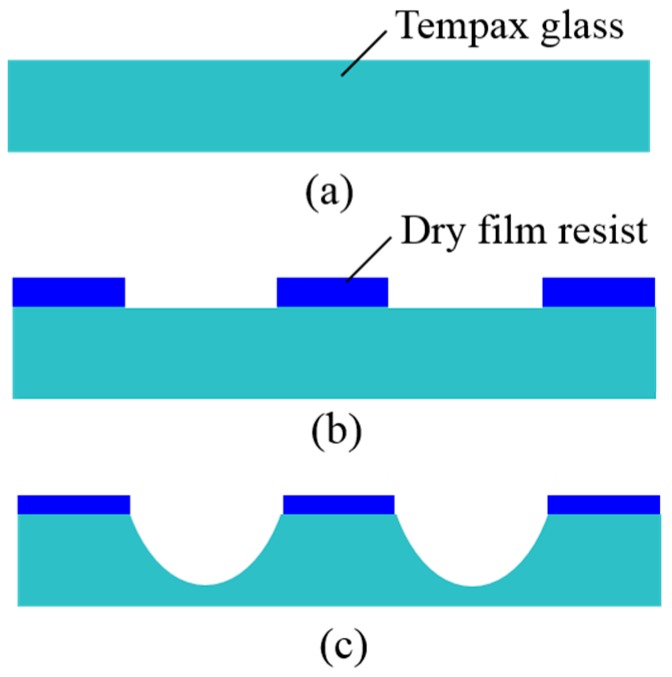
Fabrication process. (**a**) Tempax glass; (**b**) photolithography; (**c**) sandblast.

**Figure 3:**
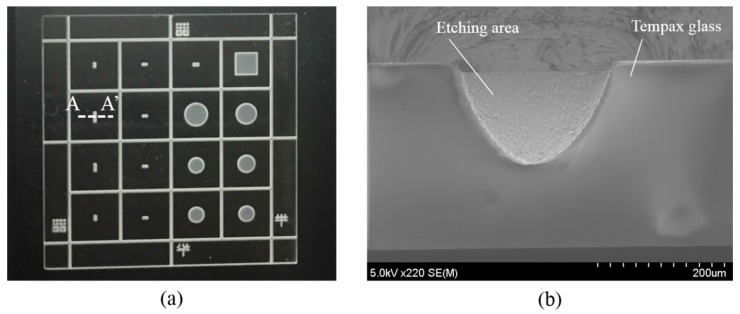
Illustration images for sandblast results. (**a**) Sandblast result on 2 cm × 2 cm Tempax glass wafer; (**b**) A-A′ cross session.

Figure 1 illustrates the sandblasting setup, which commonly consists of a nozzle, a micro-powder and a movable stage. The particles are accelerated towards the sample with high pressure airflow through the nozzles of sandblaster. The etching rate of sample is controlled by the jet velocity of powder commonly at 80–290 m/s and a movement velocity of stage (X velocity and Y velocity). In this work, the sandblasting process is used for the patterning of the Tempax glass, using Al_2_O_3_ powder with a granule size of 14 μm. Figure 2 illustrates the experimental process of the sandblast on the glass wafer. The 300-µm-thick glass substrate (Figure 2a) is employed for this process. A dry film resist (MS 7050, Toray, Tokyo, Japan) with a thickness of 50 μm is pasted, and then photolithography is performed, as shown in Figure 2b. Next, glass wafers with dry film resist patterns are etched via sandblast. A glass etching depth of approximately 150 µm was achieved under sandblasting conditions, as shown in Table 1. Illustration images for the sandblast results are shown in Figure 3. A 2 cm × 2 cm glass wafer and an A-A′ cross section are given in Figure 3a,b, respectively. Glass etching surfaces are very rough and etching profiles evolve into V-shapes, as shown in Figure 3.

In summary, the advantages of the sandblasting technique is its simplicity, low cost, and accurate directional etching; however, it is difficult for small patterns, due to the limitation of dry film resist resolution and large size of powder particles. Moreover, rough etching surfaces and taper etching profiles face difficulties as demonstrated above. The Al_2_O_3_ powder penetrated into the etching surfaces during the sandblast can be released in post-processes. Therefore, many particles may occur on the surface of device fabrication. The advantages and disadvantages of this method are compared to other methods in Table 2.

### 2.2. Wet Etching

The wet etching of glass has been investigated by many researchers [14,15]. The advantages of this method are its simplicity, high etching rate, high mask selectivity, low surface roughness, *etc*. However, due to its isotropic etching behavior, the aspect ratio is limited. A buffered HF (diluted HF (Hydrofluoric acid) with ammonium fluoride (NH_4_F)) solution is used for the etching of SiO_2_ because of the low damage to the photoresist. Therefore, the durability to the etching solution is improved. Higher etching rate can be achieved by increasing the concentration of the HF solution; however, the quality of the photoresist mask becomes poor. Thus, it may only be suitable for the etching of thin SiO_2_ layers. To overcome this problem, Cr-Au is one selection for the masking material for wet etching due to the inert property of Au when in contact with HF. The etching of glass has more difficulties than that of SiO_2_ due to its compositions. Therefore, deep etching requires long etching times. In this work, the Tempax glass was etched using a diluted solution of 50% HF:DI (deionized water) = 2:1. Summarized wet etching conditions are shown in Table 3. The chemical reaction of the glass in the HF solution is as follows:

SiO_2_ + 4HF = SiF_4_ + 2H_2_O (1)

**Figure 4:**
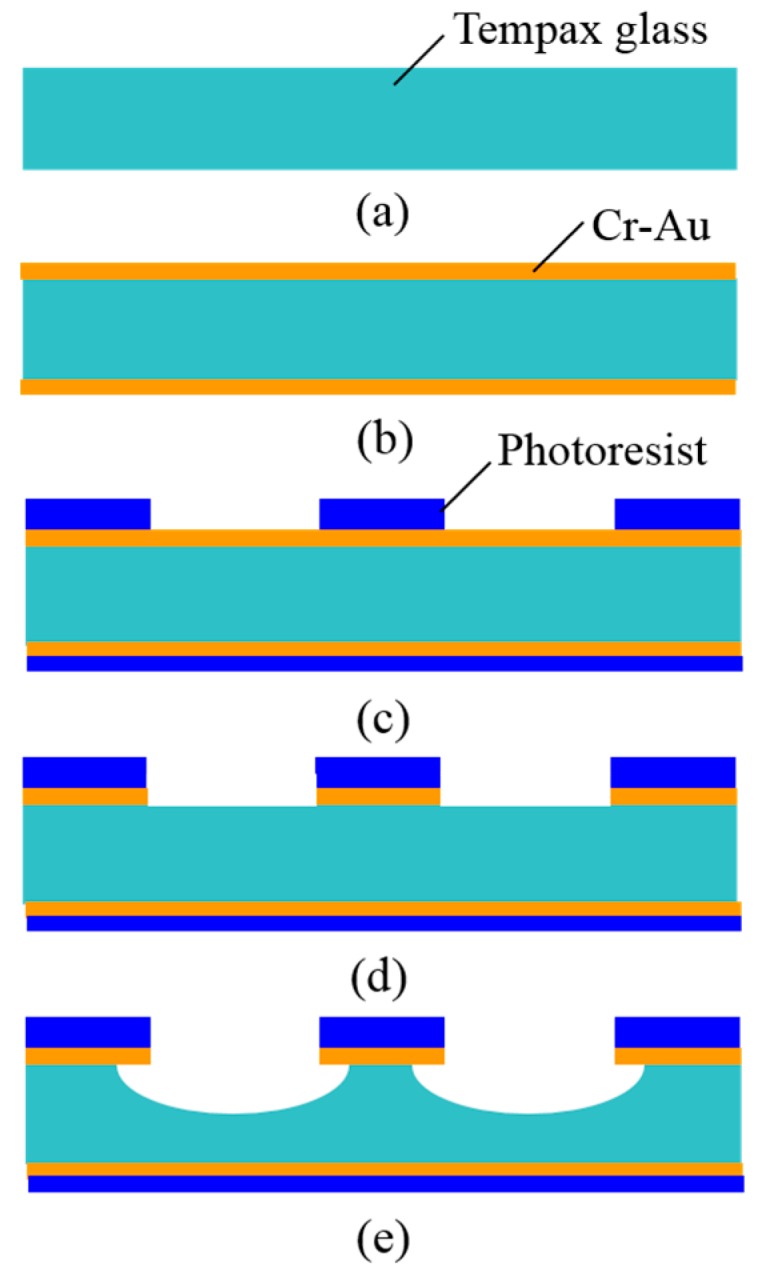
Wet etching process. (**a**) Tempax glass; (**b**) Cr-Au sputter; (**c**) photolithography; (**d**) Cr-Au wet etching; (**e**) glass wet etching.

**Figure 5:**
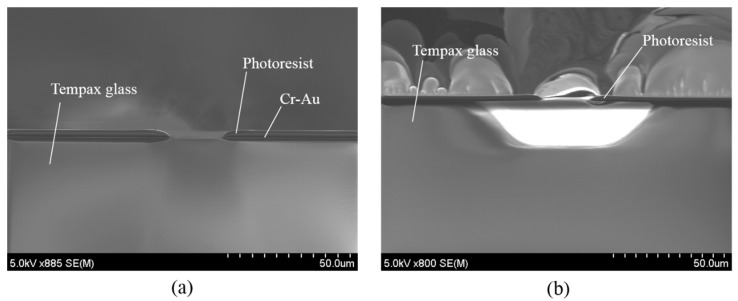
(**a**) Photolithography and metal etching; (**b**) glass etching result.

The wet etching process starts from a 300-µm-thick glass substrate (Figure 4a). 30-nm-thick Cr and 300-nm-thick Au layers are deposited on both sides of the Tempax glass wafer via sputtering (Figure 4b). Conventional photolithography, using a photoresist (OFPR 200 cp), is performed on the front side to make the mask pattern. The same photoresist is coated on the back side (Figure 4c). Then, Cr-Au layers are etched by the wet etchant (Figure 4d). The experimental result is shown in Figure 5a. Finally, the wafer is dipped in the etching solution of the diluted HF (Figure 4e). The glass structure with an etching depth of approximately 20 μm was achieved by the above solution and an etching time of 10 min. The side etching of the glass with a length of about 20 μm was observed as shown in Figure 5b.

In summary, the wet etching of glass is one of the simplest methods for glass micromachining; however, the patterning glass structure is not precise due to isotropic etching behavior. The advantages and disadvantages of this method are compared to other methods in Table 2.

### 2.3. Reactive Ion Etching

There are very few studies that mention deep glass etching together with smooth surfaces, vertical sidewalls, and high-aspect-ratio structures. The aspect ratio of a fabricated glass structure is limited because of etching anisotropy and low selectivity between glass and mask materials. Deep glass etching has been presented in the literature [16,17,18], but surface roughness and a low aspect ratio have been reported. The deep glass etching together with smooth surfaces, vertical shapes, and high aspect ratio are still difficult for micro-fabrication until now.

A laboratory-made RIE equipment [23] is employed for the deep Tempax glass etching. The RIE instrument is one kind of a magnetron-type RIE. Plasma is generated by supplying a 13.56 MHz RF generator and connected to the cathode. The cathode is made out of aluminum with an 80-mm diameter and separated from a grounded circular cooling system by a Teflon substrate to reduce stray capacitance. A samarium-cobalt (Sm-Co) permanent magnet placed on the top glass cover is employed to generate high-density plasma. The internal diameter of the etching chamber is 145 mm, and the gap distance between the top glass cover and the stage is 13 mm. A turbo molecular pump (TMP) with a pumping speed of 300 L/s is used to evacuate the chamber during the etching process, and it helps to reduce the significant re-depositing of reaction products on the sample surface. Samples are attached to the cathode stage using silicone grease for heat conduction to the stage.

**Figure 6:**
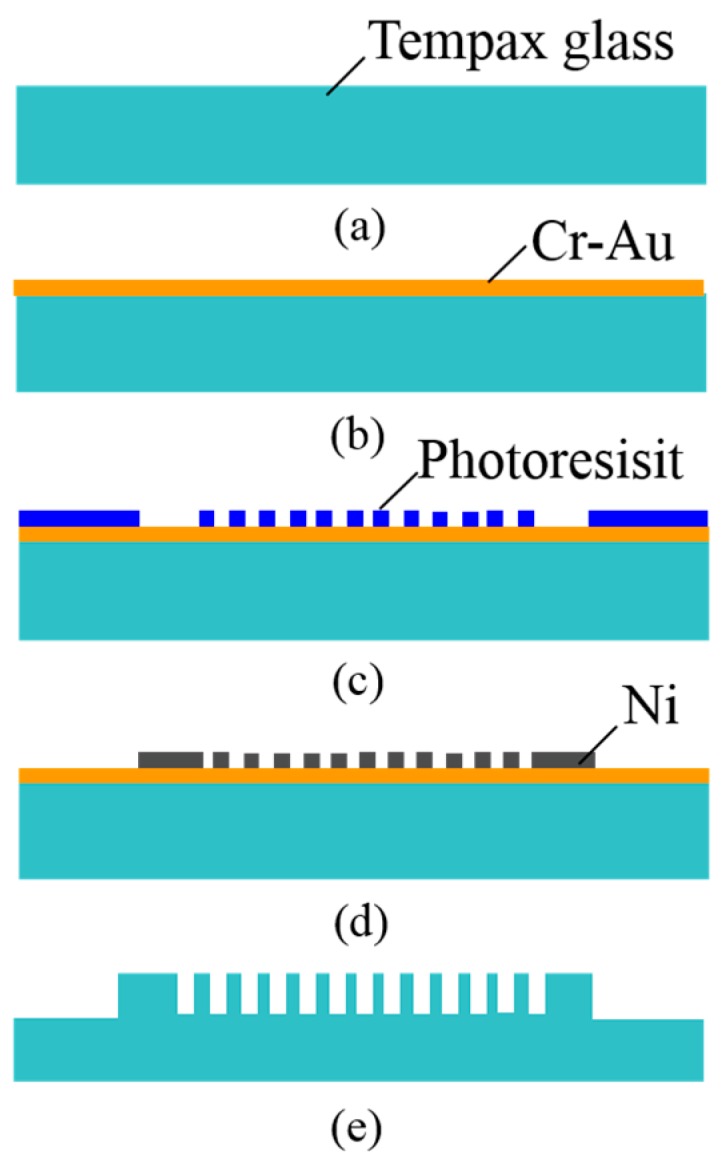
Fabrication process. (**a**) Tempax glass wafer; (**b**) Cr-Au sputtering; (**c**) immersion photolithography; (**d**) nickel electroplating; (**e**) RIE process.

The 300-μm-thick Tempax glass wafer (Figure 6a) was used for this investigation. Cr-Au was used as a seed layer for electroplating with thicknesses of 10 nm and 40 nm, respectively. They were deposited on the Tempax glass wafer via sputtering (Figure 6b). A 1-μm-thick positive photoresist (TSMR V90, Tokyo Ohka Kogyo, Kanagawa, Japan) was then coated on the Cr-Au films and patterned via immersion lithography (Figure 6c). A mold formed by the photoresist film was filled with nickel via an electroplating method. The 600-nm-thick nickel was formed on the Cr-Au surface (Figure 6d). Next, the photoresist was removed with a resist stripper (MS-2001), and the Tempax glass was etched out via the RIE process using a mixture gas of SF_6_ and O_2_.

A high glass etching rate (more than 300 nm/min) and high etching mask selectivity (more than 30) were achieved by using a gas mixture of SF_6_ at 40 sccm and O_2_ at 4 sccm, RF power at 100 W and gas pressure at 0.25 Pa. The self-bias voltage of −380 V was generated under those etching conditions. Summarized RIE conditions are shown in Table 4.

**Figure 7:**
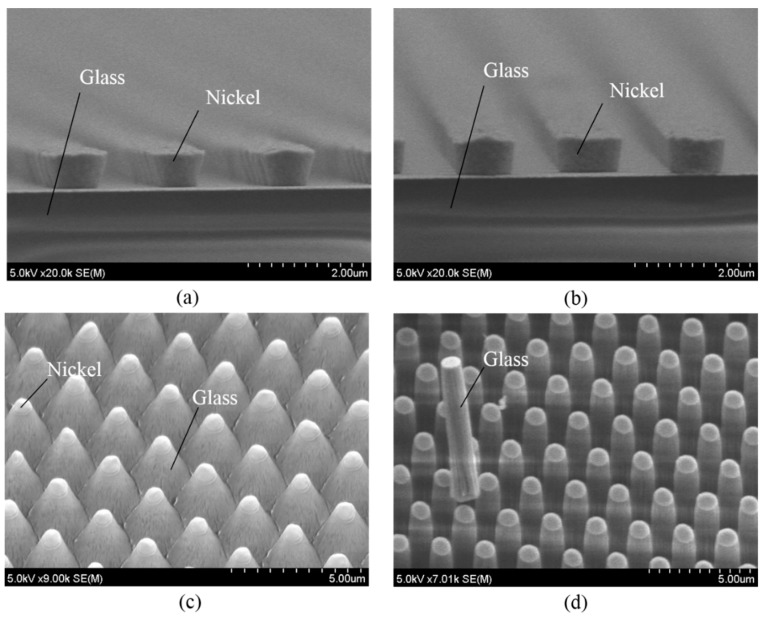
Experimental results. (**a**) Tilted mask profile (mask #1); (**b**) vertical mask profile (mask #2); (**c**) tapered cylinder profile; (**d**) vertical cylinder profile.

The experimental processes on magnetron-type RIE of the Tempax glass substrate with different nickel mask shapes were studied. The cross-sectional images of tilted (mask #1) and vertical mask (mask #2) shape profiles are shown in Figure 7a,b, respectively. The Tempax glass wafer was etched out under the same conditions mentioned above for both mask shape profiles. Firstly, RIE was performed by using mask #1. The RIE etching result indicated a tapered cylinder shape (base angle ~80°) and the 30°-tilted SEM (scanning electron microscope) image of the etched profile is shown in Figure 7a. The etched profile in the case of mask #2 is shown as a vertical shape (base angle ~89°) in Figure 7b. Thus, glass pillars having smooth surfaces, vertical shapes and a high aspect ratio of 10, with an etched depth of 10 μm and a pillar diameter of 1 μm, were achieved. From those experiments, the mask profiles are thought to influence the etched profile due to the reflection of ions at mask side walls and the mask damage caused by the mask etching.

**Figure 8:**
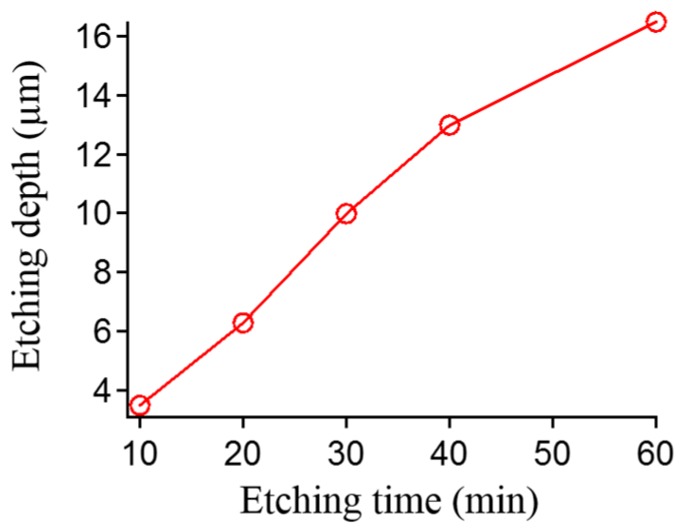
Glass etching depth as function of etching time.

**Figure 9:**
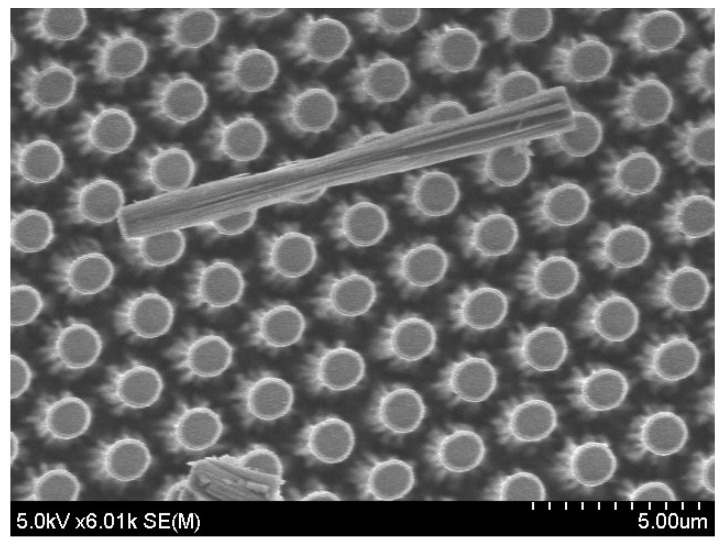
Pillars after 60-min etching process.

The thickness of nickel masks does not change; therefore, an increase in etching depth of the Tempax glass substrate is possible. The etching depth increases with progressing etching time while the etching rate slightly decreases, as shown in Figure 8. This phenomenon is called RIE-lag [24]. Higher aspect ratio of glass pillar structures can be achieved, but their surfaces become rough (Figure 9). Etching depth of glass can reach approximately 17 μm after an etching process of 60 min.

In summary, the high-aspect-ratio structure together with a smooth surface and vertical shapes of Tempax glass substrate can be achieved via the investigation of RIE. This technique is a potential candidate for micro-nano scale glass micromachining. The advantages and disadvantages of this method are compared to other methods in Table 2.

### 2.4. Glass Reflow Process

The glass structure can be formed by using the glass reflow process; nevertheless, its process faces difficulty when the glass fills into small patterns [19,20]. The fabrication of glass capillaries based on a glass reflow into a small trench has been introduced in our previous work [25]. Glass reflow into large cavities, a micro-trench and a micro-capillary is investigated in this work. At first, a description of the glass reflow process will be presented with illustrations of experimental results. Then, by using additional optimization, the glass reflow into a micro-trench and a micro-capillary is demonstrated.

**Figure 10:**
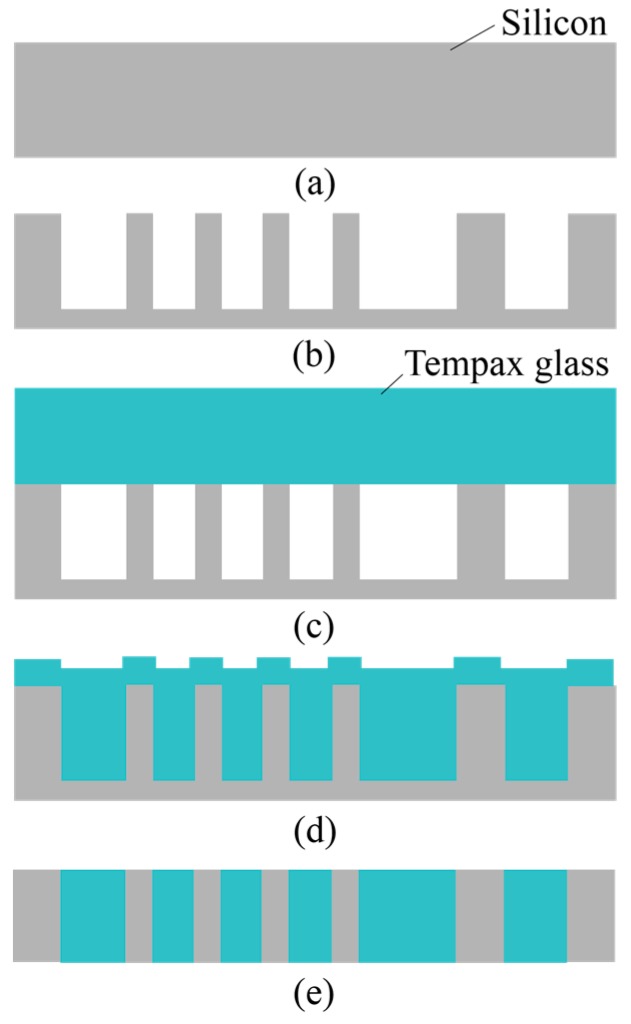
Glass reflow process. (**a**) Silicon wafer; (**b**) deep RIE; (**c**) anodic bonding in a high vacuum chamber; (**d**) glass reflow process; (**e**) CMP.

**Figure 11:**
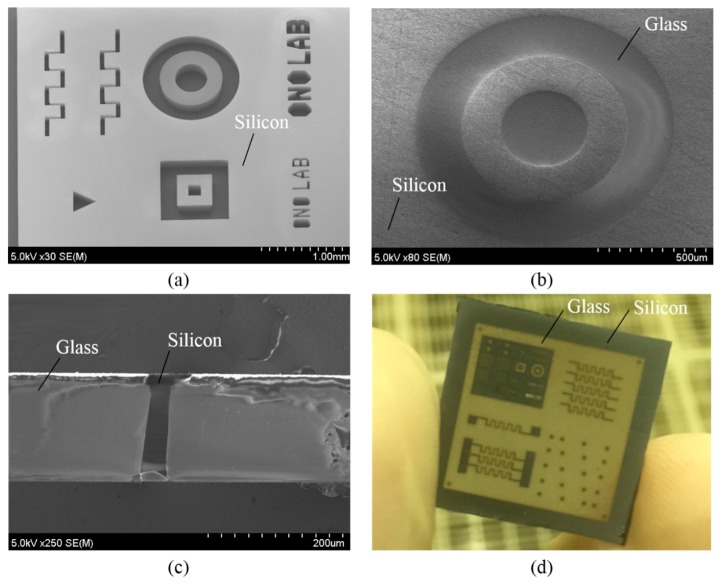
Glass reflow process. (**a**) Silicon mold; (**b**) glass reflow into silicon mold; (**c**) silicon through-glass wafer interconnects; (**d**) 2 cm × 2 cm glass-in-silicon wafer.

Figure 10 schematizes the glass reflow process. A p-type silicon wafer with a thickness of 300 μm is used as the base (Figure 10a). A 500-nm-thick SiO_2_ layer is formed on the above substrate by wet thermal oxidation under conditions of temperature at 1100 °C and a process time of 40 min. This SiO_2_ layer was employed as an etching mask of the silicon wafer. The etching resist mask was formed via photolithography, and the SiO_2_ layer was partly etched by RIE using CHF_3_ and Ar gas mixtures with a process pressure of 5 Pa and an RF power of 120 W. The silicon structures (silicon mold) were patterned by deep RIE based on Bosch process using SF_6_ and C_4_F_8_ (Figure 10b). The experimental result is shown in Figure 11a. The silicon structure with an etching depth of 230 μm was achieved.

The patterning silicon wafer and Tempax glass was bonded together at 400 °C with an applied voltage of 800 V for 15 min (Figure 10c). This process was performed in a high vacuum chamber of 0.01 Pa for eliminating air from the cavities of silicon mold. Then, the bonded silicon-glass wafer was annealed in an atmospheric furnace with a high temperature of 750 °C for 10 h (Figure 10d). Tempax glass was melted and filled into cavities of silicon mold because the transition temperature of the Tempax glass was around 550 °C. Then, both of the Tempax glass and the silicon sides of the wafer were lapped and polished via CMP (chemical mechanical polishing) (Figure 10e). The complete filling process into cavities was achieved as shown in Figure 11b–d. Thus, the glass micromachining can be done via the glass reflow process.

**Figure 12:**
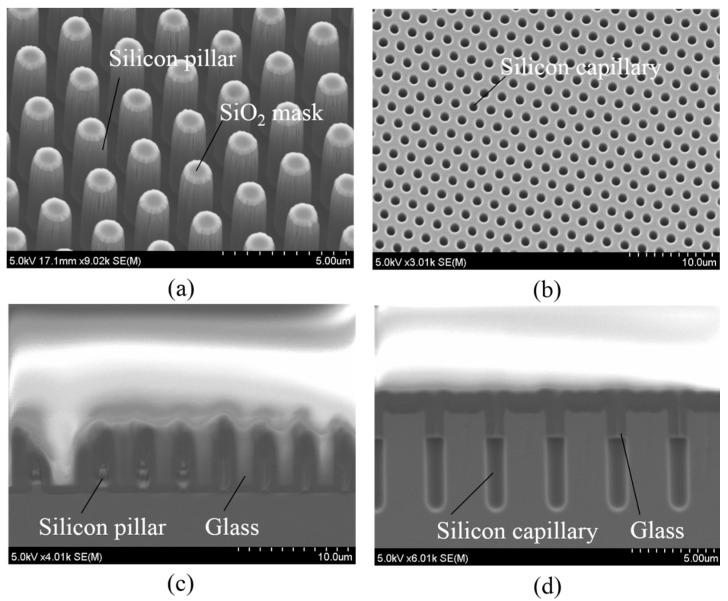
Glass reflow process into small cavities. (**a**) Silicon pillar mold; (**b**) silicon capillary mold; (**c**) penetration depth of pillar mold under third reflow condition; (**d**) penetration depth of capillary mold under third reflow condition.

Next, we present the additional optimization of the process flow for glass reflow into a micro-trench and a micro-capillary. The silicon pillar (pillar mold, Figure 12a) and capillary (capillary mold, Figure 12b) structures with the parameters in Table 5 were prepared as the silicon molds. Silicon-to-glass anodic bonding was performed in the same conditions as above. Then, we perform the reflow conditions of the process flow as shown in Table 6. The glass could be filled into the silicon pillar mold with the penetration depth of 1.5 μm, while that of the silicon capillary mold experienced almost no penetration under the first reflow condition at 1000 °C and process time of 3 h. The penetration depth was considered a function of process time. A long reflow process time was performed; nevertheless, a complete fill into the both pillar and capillary molds has not yet been achieved. Only 5-μm-thick glass can be penetrated into pillar mold, and 1-μm-thick glass can be filled into capillary molds under the second reflow condition at 1050 °C and process time of 16 h. Adequate penetration into silicon molds was not achieved in the above conditions (first and second reflow conditions). The main reasons are due to process time, low vacuum cavities, and low surface wettability against the glass. Thus, optimization conditions are needed to solve the problem.

A thin SiO_2_ film with a thickness of 50 nm grown on the surface of a silicon mold via dry thermal oxidation before anodic bonding process was proposed. The merits of this SiO_2_ can enhance the surface wettability against the glass; therefore, the glass can very easily penetrate small cavities such as the pillar and capillary molds. Moreover, the organic materials or passivation layer of silicon molds due to the deep RIE process can be removed because of the high temperature process of thermal oxidation. Therefore, the lower pressure level of silicon molds can be achieved after sealing process. The third reflow condition at a high temperature of 1100 °C with a long process time of 20 h was performed. The complete filling process into the pillar mold was observed while only 2.5-μm-thick glass could penetrate the capillary mold. The experimental results under the third reflow condition for pillar and capillary molds are shown in Figure 12c,d. Thus, glass reflow into micro-capillary molds is more difficult than that into micro-pillar mold. The possible reasons are due to small space and high surface tension of the micro-capillary mold. A longer reflow process time may be one of the solutions to an adequate fill into this mold.

In summary, glass structures were formed using a silicon mold with a high-temperature environment, a long process time, and an assistance of enhancement of the surface wettability (a thin SiO_2_ layer). Glass reflow into large cavities, micro-trench, and micro-capillary were thus demonstrated. The advantages and disadvantages of this method are compared to other methods in Table 2.

## 3. Conclusions

Four techniques for glass micromachining, including sandblast, wet etching, RIE, and glass reflow techniques, were demonstrated in this paper. The advantages, together with the disadvantages, of each method were presented and discussed in light of the experiments. Sandblast and wet etching techniques are simple processes, but they are facing difficulty in small and high aspect ratio structures. A sandblasted 2 cm × 2 cm Tempax glass wafer with a depth of approximately 150 µm was presented, and the rough etching surfaces and V shape profiles were observed. The Tempax glass structure with an etching depth and sides of approximately 20 μm was done via the wet etching technique. Depth glass pillars with a smooth surface, vertical shapes, and a high aspect ratio of 10 with a depth of 10 μm, a diameter of 1 μm, and the pitch of two pillars of 2 μm was achieved via the RIE technique. The glass micromachining was successfully demonstrated via the glass reflow technique. Glass reflow into large cavities, a micro-trench and a micro-capillary was investigated, and the additional optimization of process flow was performed for glass penetration into micro-scale patterns.

## Figures and Tables

**Table 1:** Sandblast conditions.

Etching Material	Mask Material	Nozzle Pressure	X Velocity	Y Velocity
Tempax Glass	Dry thin film resist (MS7050)	0.1 Pa	10 mm/s	20 mm/s

**Table 2:** Summarized advantages and disadvantages of glass micromachining.

Parameters	Sandblast	Wet Etching	RIE	Glass Reflow
Feature size				
Minimum size	100 μm	1 μm	<1 μm	<1 μm
Side etching	No	Yes	No	No
Etching profile	V shapes	U shapes	Vertical	Vertical
Aspect ratio	Low	Low	High	High
Surfaces	Rough	Smooth	Smooth	Smooth
Process time	Short	Short	Medium	Long
Mask materials	Dry film resist	Metal and photoresist	Metal mask for high selectivity	Silicon mold
Selectivity between Tempax glass and mask material	Low	High	High	Glass fills into cavity
Etching environment	Al_2_O_3_ particles	Liquid	Plasma	Atmospheric furnace with a high temperature
Post processes	Particles	Good	Good	Good

**Table 3:** Wet etching conditions.

Etching Material	Mask Material	Etching Solution	Etching Rate	Side Etching
Tempax Glass	Photoresist on metal (Cr-Au)	HF:DI = 2:1	2 µm/min	2 µm/min

**Table 4:** RIE conditions.

Etching Material	Mask Material	Gas	RF Power	Gas Pressure	Etching Rate
Tempax Glass	Nickel	SF_6_ & O_2_	100 W	0.25 Pa	300 nm/min

**Table 5:** Summarized parameters of pillar and capillary molds.

Molds	Diameter	Pitch	Depth
Pillar mold	1.2 μm	2 μm	8 μm
Capillary mold	1 μm	2 μm	6.5 μm

**Table 6:** Glass reflow conditions.

Parameters	1st Reflow Condition	2nd Reflow Condition	3rd Reflow Condition
Temperature	1000 °C	1050 °C	1100 °C
Process time	3 h	16 h	20 h
Mold surface	Silicon	Silicon	SiO_2_
Penetration depth of pillar mold	1.5 μm	5 μm	8 μm
Penetration depth of capillary mold	0 μm	1 μm	2.5 μm
